# Using Component-Resolved Diagnostics in the Management of Peanut-Allergic Patients

**DOI:** 10.1007/s40521-016-0080-6

**Published:** 2016-04-07

**Authors:** F. C. van Erp, R. J. B. Klemans, Y. Meijer, C. K. van der Ent, A. C. Knulst

**Affiliations:** Department of Paediatric Pulmonology and Allergology, University Medical Centre Utrecht, Utrecht, The Netherlands; Department of Dermatology and Allergology, University Medical Centre Utrecht, Utrecht, The Netherlands; Department of Paediatric Pulmonology and Allergology, University Medical Center Utrecht, Wilhelmina Children’s Hospital, P O Box 85090, 3508 AB Utrecht, The Netherlands

**Keywords:** Peanut allergy, Diagnosis, Component-resolved diagnostics, CRD

## Abstract

Instead of relying on crude peanut extract, component-resolved diagnostics (CRD) uses sensitization to allergenic proteins within peanut. In this review, we describe the recent advances and future perspectives of the use of CRD in the management of peanut-allergic patients. There is strong evidence that sensitization to Ara h 2 is the best predictor for clinically relevant peanut allergy in children and adults. Isolated sensitization to other peanut components is only rarely present in patients with systemic reactions to peanut. It is, however, important to remark that cut-off points of sIgE to Ara h 2 that predict tolerance or allergy vary between different study populations, different age groups and geographical regions, and validation studies performed in different settings are necessary to implement cut-offs in daily practice. Future studies should focus on the role of CRD in risk-assessment early in life, predicting long-term tolerance and monitoring treatment responses following immunotherapy.

## Introduction

Peanut is one of the most common allergens capable of eliciting severe allergic reactions [1]. Moreover, peanut allergy can already appear during early childhood and often persists throughout life [2••]. Depending on the geographical region studied and definition of allergy used, peanut allergy is estimated to affect 0.2–3 % of the population [3, 4]. Peanut allergy is suspected when immediate allergic symptoms occur after peanut ingestion together with positive sensitization. Sensitization to peanut can be detected by a raised level of specific IgE (sIgE) or positive skin prick test (SPT). Sensitization is not always accompanied with clinical reactivity. The gold standard to diagnose peanut allergy is a double-blind placebo-controlled food challenge (DBPCFC) [5]. However, the DBPCFC is a burdensome, expensive and potentially dangerous procedure and therefore alternative ways to predict peanut allergy are strongly required [6]. In addition, previous work indicated that (double-blind) food challenges can be false-negative and are subject to observer variability, especially when objective symptoms are absent [7, 8].

In recent years, the role of sIgE to peanut components in the diagnostic work-up of patients with suspected peanut allergy has been extensively studied. Instead of relying on crude peanut extract, component-resolved diagnostics (CRD) uses sensitization to purified or recombinant allergenic proteins within peanut. CRD has proven to strongly increase the diagnostic accuracy to test for peanut allergy. Moreover, it is able to identify cross-reactivity and has the potential to classify patients at higher risk for systemic reactions [9]. Moreover, reactivity to individual peanut allergens might be able to predict resolution of peanut allergy and be a target for immunotherapy [10•, 11].

In this review we describe the recent advances and future perspectives of the use of CRD in the management of peanut-allergic patients.

## Peanut extract and components

Peanut (*Arachis hypogaea*) belongs to the botanical family Fabaceae which is also known as Leguminosae and commonly known as the bean or pea family. The protein content of peanut lies between 24–29 % and is mostly made of seed or storage proteins [12].

Currently, 17 allergens (components) of peanut (Ara h 1–17) have been identified in the official allergen nomenclature database [13]. Only the first nine of those allergens have been studied in relation to peanut allergy in humans and will, therefore, be part of this review. These allergens belong to the cupin (Ara h 1, 3), conglutin or prolamin (Ara h 2, 6, 7), profilin (Ara h 5), Bet v 1 homologous proteins or pathogenesis-related proteins of class 10 (PR-10) (Ara h 8) or lipid transfer protein (Ara h 9) family. The allergens can also be divided in more functional and clinically relevant categories: storage proteins, pollen-associated proteins and plant pan allergens. The characteristics of peanut allergens are shown in Table 1.

**Table 1:** Characteristics of currently characterized relevant peanut allergens

Name	Protein family	Availability	Stability	% of total protein	Type	Cross-reactivity	Related proteins
Ara h 1	Cupin 7S globulin	ISAC/CAP	+++	12–16 %	Storage	+	Gly m 8 Cor a 11
Ara h 3/4^a^	Cupin 11S globulin	ISAC/CAP	+++	38–76 %	Storage	+	Cor a 9 Gly m 9
Ara h 2	Conglutin 2S albumin	ISAC/CAP	+++	5.9–9.3 %	Storage	+	Cor a 14 Gly m 8
Ara h 5	Profilin	–	–	*No data*	Pollen-associated	+++	Bet v 2 Gly m 3
Ara h 6	Conglutin 2S albumin	ISAC	+++	4–14 %	Storage	+	Cor a 14 Gly m 8
Ara h 7	Conglutin 2S albumin	–	*No data*	0.5 %	Storage	*No data*	Cor a 14 Gly m 8
Ara h 8	PR-10	ISAC/CAP	–	< 0.1 %	Pollen-associated	+++	Bet v 1 Mal d 1
Ara h 9	Lipid transfer protein	ISAC/CAP	++	*No data*	Plant pan allergen	+++	Pru p 3 Art v 3

*Bet* birch pollen, *Gly* soy, *Pru* peach, *Cor* hazelnut, *Art* mugwort, *Ses* sesame

^a^Ara h 4 shares 91.3 % nucleotide sequence homology with Ara h 3 [14]

References used for this table [9, 13, 15•, 16]

### Storage proteins

Seed storage proteins Ara h 1, 2, 3, 6 and 7 have a high degree of thermal and digestive stability [17]. As a result, they are the major peanut allergens. Ara h 1 is a 7S globulin and is recognized in 26-92 % of peanut-allergic patients [18–22, 23••, 24, 25•]. Between 20 and 80 % of peanut-allergic patients are sensitized to the 11S globulin Ara h 3 [18–22, 23••, 24, 25•]. Ara h 3 and Ara h 4 are isoforms of each other and considered to be the same allergen [26]. Ara h 2, 6 and 7 belong to the 2S albumin protein family and have a high amount of amino acid sequence identity [27]. Ara h 2 and Ara h 6 are considered as the most potent allergens and are recognized by the majority (60–100 %) of peanut-allergic patients in Western Europe and the USA [19, 20, 22, 24, 28]. However, only up to 60 % of the Mediterranean peanut-allergic patients show raised levels of sIgE to Ara h 2 [23••, 29].

### Bet v 1 homologous protein

Ara h 8 is an allergen of the PR-10 family and has low stability to roasting and digestion. PR-10 allergens are common pan allergens in pollens and also present in vegetables and fruits. Due to cross-reactivity with the birch pollen allergen Bet v 1, sensitization to Ara h 8 is common especially in North-West Europe [30, 31••]. Furthermore, Ara h 8 is also cross-reactive with Gly m 4 from soy and potentially with white lupine [32, 33].

### Lipid transfer proteins

Ara h 9 has been identified as an important lipid transfer protein (LTP) allergen in peanut, especially in the Mediterranean area [31••, 34]. LTPs are very stable and LTP-sensitized patients can experience systemic allergic reactions in addition to oral allergy. A strong association between sensitization to the LTPs in peach (Pru p 3) and peanut in Spain has been described [35]. Besides Pru p 3, it has also been suggested that LTP from plane tree (Pla a 3) or mugwort (Art v 3) can act as primary sensitizers [36, 37].

## How to use CRD: in the diagnosis of peanut allergy

There is strong evidence that sIgE to Ara h 2 is the best predictor for peanut allergy in children and adults [38••]. Depending on the population studied and definitions used, sensitivity ranges from 60–100 % and specificity from 60 to 96 % when using a cut-off of 0.35 kU/L [18–22, 24, 39–41]. The best combination of positive and negative likelihood ratio was also found when using sIgE to Ara h 2. Although it has been suggested that the prevalence and relative importance of sIgE to Ara h 2 is lower in Mediterranean countries, sIgE to Ara h 2 also emerged as the best predictor in studies from Southern France and Spain [20•, 26].

In daily practice, sIgE to Ara h 2 and peanut extract are both suitable to exclude peanut allergy [38••]. However, sIgE to Ara h 2 is more specific and sensitive to diagnose peanut allergy. An 80–95 % NPV was reached when using sIgE to Ara h 2 levels of <0.35 kU/L and a 100 % NPV when a cut-off level of <0.1 kU/L was used [28, 42]. Moreover a 95–100 % PPV was reached when using sIgE to Ara h 2 levels >5 kU/L to diagnose peanut allergy [28, 40]. By using optimal cut-off points for sIgE to Ara h 2 (i.e. with the highest NPV and PPV) peanut allergy could be diagnosed without a food challenge in the majority of subjects suspected of peanut allergy. However, it should be noticed that current available cut-off points were estimated in a selected group of referred patients and, therefore, cannot be generalized to other centres without validation studies. Furthermore, data in adults and young children (<4 years) are currently lacking.

It should also be kept in mind that in some cases of peanut allergy, other peanut components are relevant [43•, 44•]. Allergic patients without sIgE to Ara h 2 but with Ara h 1 or Ara h 3 sensitization have been reported occasionally [31••, 42]. The sensitivity of sIgE to Ara h 1 and Ara h 3 is generally low but varies extensively between studies (26–92 % and 21–84 %), mainly depending on the geographical region [38••]. Two studies in children and adults describe that the diagnostic accuracy of sIgE to Ara h 6 is comparable to sIgE to Ara h 2 [23••, 39]. This can be explained by the homology and cross-reactivity between these two 2S albumins [27]. In adults, it was advocated that Ara h 6 could have additional value to Ara h 2 in individual cases with a strongly suspected peanut allergy in which sIgE to Ara h 2 was absent or very low [25•, 42]. The diagnostic value of sIgE to Ara h 8 is low with a sensitivity ranging from 16 to 42 % and specificity from 31 to 100 %. Isolated Ara h 8 sensitization is often related to Bet v 1 sensitization and associated with tolerance or mild local symptoms [30, 45]. In a Mediterranean region, Ara h 9 can detect LTP-related peanut sensitization; however, the added value of sIgE to Ara h 9 is questionable as cases of peanut allergy with isolated Ara h 9 sensitization are rare [25•, 42].

In summary, sIgE to Ara h 2 is the best diagnostic test to diagnose or exclude a possible peanut allergy. In case of a suspected peanut allergy and absence of sIgE to Ara h 2, additional peanut components can be determined to detect relevant other sensitization. In older children and adults with a suspected Bet v 1 related peanut allergy sIgE to Ara h 8 can be useful. In adults and children with highly suspected primary peanut allergy sensitization to other storage proteins (Ara h 1, h 3 and h 6) can be relevant.

## How to use CRD: in the prediction of severe peanut allergy

We concluded that sIgE to Ara h 2 could reduce the number of food challenges. However, next to diagnostic purposes, food challenges are used to provide useful information regarding the severity of peanut allergy and subjective and objective eliciting doses.

The severity of allergic symptoms during challenge correlated with higher levels of Ara h 2 in several studies [19, 25•, 46]. Furthermore, higher levels of sIgE to Ara h 2 were associated with lower thresholds during food challenges in children and adults [19, 47]. However, contrasting results and large individual variation in the relation between Ara h 2 and severity of peanut allergy exist [19, 48]. There are several explanations for the absence of a strong and consistent association between sIgE to Ara h 2 and severity of peanut allergy. Firstly, challenges can underestimate severity of peanut allergy because they are usually stopped when objective and not necessarily severe symptoms occur. Secondly, in contrast to daily life, patients are in a relative stable situation during challenge (absence of co-factors like active allergic disease, infections or exercise) [48, 49]. In addition, it was suggested that the correlation between sIgE to Ara h 2 and thresholds only applies to higher dose therefore to selected patient populations only [19].

sIgE to the storage components Ara h 1, Ara h 3 and Ara h 6 have also been related to severity but the best correlation was found for Ara h 2 [19, 25•]. As is mentioned before, isolated sIgE to Ara h 8 is often related to mild symptoms. It has been reported that allergic reactions can occur in rare cases if a large amount of peanut is eaten over a short period of time [44•]. Furthermore, Ara h 8 is able to activate basophils in monosensitized children, and a recent report shows that natural Ara h 8 from roasted peanuts has a reasonable degree of proteolytic and thermal stability [50, 51].

In summary, severe peanut allergy is unlikely without IgE to any of the seed storage proteins Ara h 1, 2, 3 or 6. Although Ara h 2 is correlated to severity, we cannot use the level of sIgE to Ara h 2 or other components to classify individual patients at higher risk for severe allergic symptoms during challenge or in daily life.

## Other aspects of CRD

Despite the promising results of CRD in diagnosing and excluding peanut allergy, there are several important aspects of CRD that have to be considered when using and interpreting CRD in daily practice.

### Singleplex versus multiplex

Besides determination of sIgE to individual components (singleplex) with the ImmunoCAP method (Thermo Fisher, Uppsala, Sweden), it is also possible to simultaneously determine sIgE to a large number of components by the use of biochip technology (multiplex) like the ImmunoCAP ISAC (Thermo Fisher, Uppsala, Sweden). The multiplex assay requires less blood and allergen and facilitates the identification of (cross-reactive) sensitization patterns [52•]. Several studies compared the singleplex and multiplex method for peanut allergens and showed high correlation between the two methods [53, 54]. However, it has to be considered that the multiplex method has potentially lower analytical sensitivity [23••]. Furthermore, the ISAC requires manual procedures and results are semi-quantitative (expressed in standardized units). In general, multiplex CRD should, therefore, be used to investigate complex cases like patients with multiple food allergies, idiopathic anaphylaxis or severe allergic symptoms without sensitization to Ara h 2 and not as a primary diagnostic test [55].

### Availability of allergens

One of the limitations of the use of whole peanut extract in sIgE testing is that the conventional extracts vary in composition and are deficient in some IgE components [56]. However, relevant sensitization can still be missed when using CRD because some peanut allergens related to (severe) allergy might not have been identified. Moreover, some allergens that were characterized are not yet commercially available or investigated in clinical studies (Ara h 5, Ara h 7) or are only available in multiplex tests (Ara h 6), see also Table 1.

### Variability in CRD pattern within and between patient groups

The diagnostic value of different components is affected by several patient related factors. Age dependency of sensitization patterns was described. Several studies demonstrate that older peanut-allergic patients were more often sensitized to Ara h 8 in contrast to children with early onset allergy that recognize predominately Ara h 2 and to a lesser extent Ara h 1 and h 3 [31••, 57, 58]. Additionally, geographical variation in sensitization patterns presumably due to differences in exposure to other plant allergens, dietary habits and genetics [31••, 34]. Although sIgE to Ara h 2 seems accurate in diagnosing and excluding peanut allergy in different parts of the world, cut-off points may vary between countries. As mentioned before, additional components may play a role in certain regions (like Ara h 9 in Mediterranean countries).

### Validation studies

Prospective validation studies that include follow-up to detect false-negative (e.g. allergic reactions in patients with undetectable sIgE to Ara h 2) or false positive tests (negative challenges despite high levels of sIgE to Ara h 2) are needed. Those studies are necessary to further confirm the added value of CRD in daily practice and determine cost-effectiveness. Furthermore validation of CRD in other settings (e.g. secondary care), young children and in different regions of the world is necessary as the diagnostic accuracy and therefore cut-off points of a test vary with the pre-test probability of disease.

## Future perspectives of CRD

### Risk-assessment early in life

The Learning About Peanut Allergy study showed that early oral introduction of peanuts was able to successfully prevent allergy in high-risk infants [59]. Peanut avoidance was associated with an increase in peanut wheal size and a higher proportion of patients with high levels of sIgE to peanut. At this moment it is unknown whether CRD in those very young children can be used to predict an increased risk for (severe) peanut allergy and is useful in deciding which children should introduce peanut early in life.

### Predicting development of tolerance

Results from the population-based Health Nuts Study showed that in 22 % of children with positive challenge in their first year of life outgrew their peanut allergy at year four [2••]. Like in several other studies, an increased SPT and sIgE response to peanut indicated persistent peanut allergy [60–62]. However, sIgE to Ara h 2 at year one was not predictive for persistence of peanut allergy [2••]. Further studies are required to investigate whether the course of sIgE to Ara h 2 over time is related to resolution or persistence of peanut allergy.

### Selection of patients for food challenges

The DBPCFC is the current gold standard to diagnose peanut allergy but burdensome and expensive, partly because the test takes 2 or even 3 days (open challenge). Based on validated cut-off points, sIgE to Ara h 2 can be used to reduce the amount of food challenges or food challenge days. For example, patients with sIgE to Ara h 2 above the cut-off point with a 100 % PPV (i.e. 5 kU/L) are considered to have peanut allergy in our centre. If objectification of symptoms and more information about the threshold and severity is necessary, it could be speculated that in these patients the DBPCFC can be replaced by a single-day verum challenge. Furthermore, in patients with a low suspicion of peanut allergy and absence of sIgE to Ara h 2, clinical reintroduction (i.e. an open challenge with peanut butter) or even introduction at home can be advocated. It should be investigated whether certain diagnostic strategies are safe and associated with reduced health care costs and improved quality of life.

### Usefulness of components in other diagnostic tools

Peanut components Ara h 1, Ara h 2, Ara h 3 and Ara h 8 have been investigated in the basophil activation test (BAT) [50, 63, 64]. These results indicated that the basophil response to peanut components was related to clinical relevant peanut allergy and might have a higher PPV compared to sIgE to peanut components. However, more data is necessary and several practical limitations (absence of a standardized protocol, fresh whole blood samples are necessary, high costs) prevent current implementation of the BAT in daily practice [65]. Furthermore, in our recent study, we could not confirm the added value of the BAT in predicting severe peanut allergy (manuscript submitted). Skin prick tests (SPT) with peanut components were performed in the past and indicated the relative importance of Ara h 2 and Ara h 6 compared to Ara h 1 and Ara h 3 [66]. Furthermore, a correlation between the number of components detected in the skin prick test correlated with severity of peanut allergy was found on group level [67]. Although results are promising, the future of the SPT as a diagnostic test is questionable as the SPT is prone to observer (measurement and interpretation of wheal size), device and extract variability. Furthermore, tight regulations have made the production of extracts problematic [68].

### Relation to treatment response

Patients responsive to oral and sublingual immunotherapy had lower sIgE levels to peanut components Ara h 1, Ara h 2 and h 3 at baseline and at the end of studies while IgG4 binding increased at the same epitopes [10•, 69, 70]. In those studies, cut-off points of baseline Ara h 2 with 65–70 % sensitivity and 90 % specificity have been published that could predict (long-term) responsiveness to immunotherapy. However, the added value of sIgE to Ara h 2 was debatable as sIgE to peanut extract had comparable discriminative capacity. At this moment further studies are required to determine whether CRD is able to select patients for immunotherapy and predict the long term outcome of treatment strategies.

## Conclusion

The highlights of this review are presented in Table 2. In summary, we can conclude that CRD plays an essential role in the diagnostic evaluation of a patient with suspected peanut allergy. sIgE to Ara h 2 is the best predictor for peanut allergy and is preferred as first diagnostic step. Clinicians should be aware that cases of (severe) peanut allergy, sIgE to other peanut components (Ara h 1, Ara h 3, Ara h 6, Ara h 8 and Ara h 9) may be relevant. At this moment, CRD cannot be used to predict the risk of a severe allergic reaction in individual patients. The role of CRD in predicting long-term tolerance early in life and treatment response deserves further investigation.

**Table 2:** The role of CRD in the management of peanut-allergic patients

CRD as diagnostic tool	• Sensitization to Ara h 2 is the best predictor for clinically relevant peanut allergy in children and adults. • Cut-off points of sIgE to Ara h 2 that predict tolerance or allergy vary with age, geographical region and study populations and validation studies performed in different settings would be necessary to implement cut-offs in daily practice. • In the absence of sensitization to Ara h 2, other peanut components should be considered in select patient groups: ° Isolated Ara h 1, h 3 and h 6 sensitization ° Isolated Ara h 8 sensitization is not rare and often related to Bet v 1 sensitization and mostly associated with tolerance or mild local symptoms in older children and adults ° Especially in Mediterranean patients relevant isolated sensitization to Ara h 9 occurs in rare cases • Singleplex assays are preferred in patients presenting with suspected peanut allergy.
CRD in relation to severity	• Levels of sIgE to Ara h 2 or other components are correlated to severity but cannot be used to classify individual patients at higher risk for severe allergic symptoms.
Future perspectives	• At this moment, the role of CRD in risk-assessment early in life, predicting long-term tolerance or treatment responses is unclear.
